# Asymptomatic Duodenitis and *Helicobacter pylori* associated Dyspepsia in 2-Year-Old Chronic Malnourished Bangladeshi Slum-Dwelling Children: A Cross-Sectional Study

**DOI:** 10.1093/tropej/fmaa079

**Published:** 2020-10-25

**Authors:** Md Shabab Hossain, Subhasish Das, S M Khodeza Nahar Begum, M Masudur Rahman, Ramendra Nath Mazumder, Md Amran Gazi, Shah Mohammad Fahim, Mustafa Mahfuz, Rashidul Haque, William A Petri, Shafiqul Alam Sarker, Tahmeed Ahmed

**Affiliations:** 1 Nutrition and Clinical Services Division, International Centre for Diarrhoeal Disease Research, Bangladesh (icddr,b), Dhaka 1212, Bangladesh; 2 Department of Pathology, Bangladesh Specialized Hospital, Dhaka 1207, Bangladesh; 3 Department of Gastroenterology, Dhaka Medical College and Hospital, Dhaka 1000, Bangladesh; 4 Division of Infectious Diseases and International Health, University of Virginia, Charlottesville, VA 22908, USA

**Keywords:** Bangladesh, child, duodenitis, dyspepsia, *Helicobacter pylori*, malnutrition

## Abstract

**Aim:**

There is insufficient knowledge on the * duodenal histology and *Helicobacter pylori* infection in malnourished Bangladeshi children. Therefore, we attempted to explore the prevalence of *H. pylori* infection and duodenal histopathology in 2-year-old chronic malnourished Bangladeshi slum-dwelling children and investigate their association with dyspeptic symptoms.

**Methods:**

This cross-sectional study was conducted using the data of the Bangladesh Environmental Enteric Dysfunction study in an urban slum of Dhaka, Bangladesh. With a view to address the association of environmental enteric dysfunction (EED) with stunting, upper gastrointestinal endoscopy was performed on 54 chronic malnourished children {31 stunted [length-for-age *Z*-scores (LAZ) <−2] and 23 at risk of stunting (LAZ <−1 to −2)} aged between 12–24 months and the mucosal biopsies were subjected to histopathological examination after obtaining proper clinical history. Stool antigen for *H. pylori* (HpSA) was assessed to determine *H. pylori* status.

**Results:**

In all, 83.3% (45/54) of the children had histopathological evidence of duodenitis. Chronic mild duodenitis was found to be the most prevalent form of duodenitis (53.7%) in the children. Only 8.9% (4/45) of the children with duodenitis had dyspepsia (*p* < 0.05). The 14.8% (8/54) of the children were found positive for *H. pylori* infection. Logistic regression analysis revealed children positive for HpSA had significant association with dyspepsia (OR 9.34; 95% CI 1.54–56.80).

**Conclusions:**

The number of chronic malnourished children suffering from duodenitis was found to be very high. Majority of these children was asymptomatic. Children positive for HpSA had significant association with dyspeptic symptoms.

## INTRODUCTION

The term ‘dyspepsia’ is derived from Greek words ‘dus’ meaning ‘bad’ and ‘peptien’ meaning ‘to digest’ [1]. This term is widely used as a shorthand description for an array of symptom patterns attributed to disorders of the upper alimentary tract. Thomson *et al.* [2] described the term dyspepsia as the subjective reporting of symptoms that originate from the upper gastrointestinal (GI) tract. Up to 8–10 years of age these subjective symptoms are limited. So, it is evident that dyspepsia in 2-year-old children is so frequently hidden due to the misleading symptoms that it may pass almost entirely unnoticed [3], resulting in increased feeding refusal that can deprive the child of essential nutrients at this vital growing phase of life, thus hindering physical, cognitive and intellectual development. Studies show that, dyspeptic children have worse nutritional status compared with healthy controls [4].

An average of 25% of the total population suffers from dyspeptic symptoms across the globe [5]. The prevalence rate varies from 8% to 41% in Bangladeshi adults [6]. However, there is paucity of data on the prevalence of dyspepsia in Bangladeshi children. Any upper GI pathology, namely peptic or duodenal ulcer, erosive esophagitis and malignancies can be a cause of dyspeptic symptoms. Chronic duodenitis is suspected as the early phase of duodenal ulcer and may also present with dyspepsia [7]. Some studies show that small intestinal bacterial overgrowth (SIBO) also has association with dyspepsia [8].

*Helicobacter pylori* infection is the most common bacterial infection in humans [9]. In developing countries, >70% of adult population are colonized with *H. pylori* and >50% of children become colonized before the age of 10 years [10]. Acquisition and colonization of *H. pylori*, both transient and persistent, occur predominantly in childhood [11]. A study done in a peri-urban village near Dhaka, based on C-Urea breath test for *H. pylori* showed 33% children of 10–15-month age group were *H. pylori* positive [12]. However, there are no reports on the prevalence in malnourished children. Studies show that [13, 14] *H. pylori* infection is associated with dyspepsia and a long-standing infection can induce a wide variety of upper GI tract diseases, even gastric adenocarcinoma.

The existence of an inflammatory condition of the duodenum in the absence of ulceration is known as duodenitis [15]. Very few studies exist that concern duodenal pathologies in pediatric population [16] and the knowledge gap is a lot wider in case of 2-year-old children. Out of 2772 children undergoing upper GI endoscopy over a 5-year period in the USA, 352 (12.7%) were diagnosed to have duodenitis [17]. Another recent study conducted in Turkey on 747 school children reported a 30.3% prevalence of duodenitis [16]. However, a thorough literature search yielded no studies on children from the South Asian region.

There is paucity of knowledge on the duodenal histopathology and *H. pylori* infection in malnourished Bangladeshi children as well as their association with dyspeptic symptoms. Data are even scarcer in case of children in their first two years of life. Because of this knowledge gap, we sought to evaluate these parameters in a cohort of 2-year-old chronic malnourished Bangladeshi slum-dwelling children.

## MATERIALS AND METHODS

### Study site and data collection

This study was a part of the Bangladesh Environmental Enteric Dysfunction (BEED) study (ClinicalTrials.gov ID: NCT02812615, which is being conducted among the residents of *Bauniabadh* slum in Mirpur, Dhaka. The BEED study is a community-based nutrition intervention study aimed to validate non-invasive biomarkers of environmental enteric dysfunction (EED) with small intestinal biopsy and better understand the disease pathogenesis. Ethical approvals of the study were obtained from Research Review Committee and Ethical Review Committee of icddr, b (protocol no: PR-16007; Version 1.03; 1 March 2016). In this study, a total of 54 Bangladeshi slum-dwelling children, 31 of whom are stunted [length-for-age *Z*-scores (LAZ) < −2] and 23 who are at risk of stunting (LAZ < −1 to −2) were enrolled. The children received nutritional intervention for 90 days consisting of one egg, 150 ml of whole milk, micronutrient sprinkles and nutritional counseling daily, 6 days a week. Anti-helminthic treatment was provided as per national guidelines. The details and overall design of the BEED study have already been published elsewhere [18]. After completion of nutritional therapy, all participants were assessed for responses and participants failing to respond to nutritional therapy, i.e. with LAZ scores still below −1, were considered as probable cases of EED. After obtaining proper clinical history from the parents and screening for any other disease, for instance tuberculosis, which might have resulted in malnutrition, upper GI endoscopy was performed. For this particular study, a total of 54 children who fulfilled these criteria were enrolled from July 2016 to November 2017. A written informed consent was obtained from the parents after explaining the aim and procedure of the study as well as upper GI endoscopy. Biopsy samples from 54 children were collected during upper GI procedures.

### Duodenal biopsy

Upper GI endoscopy procedures were performed by an expert endoscopist (M.R.B.) using Olympus GIF Type Q180Z scope under general anesthesia. Biopsy specimens were taken from the second part of duodenum using Radial Jaw™ 4 Pediatric 2.0 mm single use biopsy forceps (Boston Scientific Corporation, Marlborough, USA). The biopsy samples were immediately placed in vials containing 10% buffered formalin solution for fixation. Paraffin sections were prepared and stained by hematoxylin and eosin (H&E). All of the biopsies were reviewed by an expert pathologist (K.N.B.), blinded to the case histories.

### Definition of dyspepsia

Based on similar studies in children, dyspepsia was defined as presence of feed-associated irritability, feeding refusal, regurgitation or vomiting following feeding for at least once per week for at least 2 months [2, 19]. Clinical features were based on parental report along with their interpretation of the child’s symptoms.

### Duodenitis

Though duodenal inflammation can be secondary to various etiology, the histomorphological appearance might be the same [20]. Chronic mild duodenitis is the form which is characterized by a small increase in the number of chronic inflammatory cells, predominantly lymphocytes, in the lamina propria of the duodenal mucosa which might be associated with slight widening and flattening of villi [20], without any polymorphonuclear infiltration [21]. Chronic active duodenitis was particularly based on the presence of polymorphonuclear invasion, predominantly neutrophilic infiltration in the lamina propria of the duodenal mucosa along with marked histomorphological changes of the villi and crypts, active epithelial degeneration and regeneration with intercellular edema [22]. Subjective morphologic analysis of the mucosal surface architecture was carried out with LEICA DM 1000 LED microscope.

### Stool antigen for *H. pylori*

Stool for examining *H. pylori* antigen was collected from all subjects. Stool was analyzed for *H. pylori* antigen by enzyme-linked immune-sorbent assay using Amplified IDEIA™ Hp StAR™ (OXOID Limited, Hampshire, UK). Dual wavelength of 450/630 nm was used and specimens with absorbance values ≥ 0.15 were considered positive and specimens with absorbance values < 0.15 were considered negative, following the manufacturer’s instructions.

### Small intestinal bacterial overgrowth

Though no perfect test exists for the diagnosis of SIBO, not even small-bowel aspiration culture, hydrogen breath testing provides the simplest non-invasive and widely available diagnostic modality for suspected SIBO [23]. So, presence of overgrowth of bacteria in small intestine was determined by hydrogen breath test from breath samples taken from children using BreathTracker SC (QuinTron Instrument Company, Milwaukee, USA). A total of ten breath samples were collected per child including a 3 h fasting baseline sample followed by nine more samples after ingestion of glucose at 1 g/kg body weight diluted in 5 ml/kg of water. A single difference of ≥12 ppm of hydrogen value from fasting baseline was considered to be SIBO positive.

### Statistical analyses

Statistical analyses were performed using SPSS version 20.0 (IBM). Mean values, standard deviation (SD) and 95% confidence intervals (CI) of means were used to describe the distribution and prevalence. At first bivariate analysis was performed between dyspepsia with each individual factors; i.e. sex, stunting, children positive for stool antigen for *H. pylori* (HpSA), SIBO, chronic mild duodenitis, chronic active duodenitis and parasitic infections using Pearson χ^2^ test or Fisher’s exact test, whichever applicable. Results with a significance level at or below 0.2 were included in the multivariable logistic regression model. Finally, multivariable logistic regression was done to quantify the relation between dyspepsia and children positive for HpSA after adjusting for stunting.

## RESULTS

The mean age of the children who underwent upper GI endoscopy was 18 ± 2 months. Among them, 32 (60%) were female and 22 (40%) were male. Table 1 shows the sociodemographic characteristics of the study population.

**Table 1:** Demographic characteristics and results of the respondents

Characteristics	Mean ± SD
Mother’s age (years)	36.6 ± 9.7
Mother’s age at 1st birth (years)	18.8 ± 3.0
Total children ever born	1.98 ± 1.17
Mean age of children (months)	18 ± 2
Mother’s currently working status	*n* (%)
Yes	9 (16.7)
No	45 (83.3)
Father’s occupation	*n* (%)
Laborer	13 (24.1)
Service holder/businessman	9 (16.7)
Driver	11 (20.4)
Factory worker	6 (11.1)
Others(unemployment, retired)	15 (27.7)
Monthly family income in USD, median (IQR)	143 (119–178)

SD, standard deviation; *n*, number of respondents; USD, United States Dollar; IQR, interquartile range.

Table 2 shows that majority of the children (85%) was asymptomatic and 8 (14.8%) children were found positive for HpSA. On naked-eye inspection during upper GI endoscopy, all but one child having lymphoid hyperplasia at the second part of duodenum showed normal findings. However, microscopic examination of the histology of their biopsy samples revealed that 83% (45/54) of these children had duodenitis. Of them only 8.9% (4/45) had dyspepsia (*p* < 0.05).

**Table 2:** Summary of findings

Variables	Child, *n* (%)
Female	32 (59.3)
Stunted	31 (57.4)
Presence of dyspepsia	8 (14.8)
Presence of endoscopic pathology	1 (1.9)
Total histopathologic duodenitis	45 (83.3)
Chronic mild duodenitis	29 (53.7)
Chronic active duodenitis	16 (29.6)
Positive for HpSA	8 (14.8)
Positive for SIBO	7 (13)
Presence of parasitic infection	1 (1.9)

*n,* number of respondents; HpSA, *Helicobacter pylori* stool antigen; SIBO, small intestinal bacterial overgrowth.

**Fig. 1.**
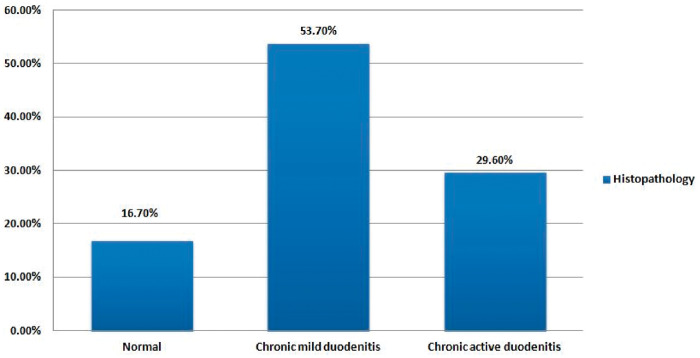
Distribution of severity of histopathology findings.

Figure 1 shows the distribution of severity of histopathology findings in study children. Chronic mild duodenitis was the most prevalent form of duodenitis in children, accounting for nearly two-thirds of all the children having duodenitis. The most notable changes in the duodenal mucosa of these children were a small increase of chronic inflammatory cells, specifically lymphocytes in the lamina propria often associated with mild degree of villous blunting. The remaining one-third of the children with duodenitis had presence of polymorphonuclear invasion, predominantly neutrophilic infiltration in the lamina propria along with histomorphological changes, active epithelial degeneration and regeneration with intercellular edema indicating presence of chronic active duodenitis.

Table 3 shows variables those are associated with dyspeptic symptoms in children. Positive HpSA was the only variable found to be significantly associated with dyspeptic symptoms (OR 9.34; 95% CI 1.54–56.80).

**Table 3:** Association of dyspeptic symptoms with HpSA

Variables	OR (95% CI)	*p*-Value*
Stunted	5.57 (0.57–54.13)	0.138
HpSA	9.34 (1.54–56.80)	0.015

HpSA, *Helicobacter pylori* stool antigen; OR, odds ratio; CI, confidence interval; *p*-value, significance level.

Although findings in the duodenum appeared macroscopically normal in almost all the children during endoscopy; their biopsy samples revealed the presence of duodenitis on histological examination. In 17% children, both the macroscopic abnormalities in endoscopy as well as histologic pathology were absent. However, both macroscopic and microscopic features of duodenitis were present in only one child.

## DISCUSSION

Our study revealed a very high prevalence of duodenitis diagnosed by histopathology in the first two years of life of chronic malnourished children living in urban slums. The finding is novel as there are no nationally representative data on duodenitis in children. A thorough literature search yielded no data from this geographic region in a similar age group. Even globally, very few studies have reported duodenal pathologies in children [16], though several studies investigated pathologies of the stomach and esophagus in the pediatric population [16]. Studies conducted in US children undergoing EGD for diagnostic purpose showed that 12.7% suffered from duodenitis [17], and the rate was 30.3% in Turkish children [16]. However, the children in those studies were well-nourished and relatively older in contrast to the chronic malnourished children in the present study. In studies conducted in western countries, the most common etiology for duodenitis was celiac disease (CD), accounting for as high as 32% [17]. In our study, no children undergoing endoscopy were found positive for CD by serum antibody test (tTG-IgA). As all the children were stunted or at risk of stunting, the etiology of duodenitis in these children might be due to EED and not due to CD *per se* [18]. Further studies assessing the biomarkers for EED in these children could establish this fact and might explain the high prevalence of duodenitis in our study population. The prevalence of CD exhibits evident racial and geographical differences, being more common in the Western population. CD in children is considered to be uncommon in the Asia–Pacific region [24]. On the other hand, EED, previously known as tropical enteropathy, is a sub-acute inflammatory condition of the small intestinal mucosa with obscure etiology accounting for more than 40% of stunting [18].

Chronic mild duodenitis was found to be the most prevalent form of duodenitis in these children. Studies show that mild inflammation of the duodenal mucosa is more frequently encountered than the other forms of duodenitis in children [20]. Routine duodenal biopsy done in a study in pediatric population yielded pathological findings in 17.4% of cases, 6.5% of which comprised of chronic mild duodenitis [25]. As majority of the children in our study was asymptomatic, this explains the high prevalence of chronic mild form of duodenitis in these children. On the other hand, active duodenitis based on polymorphonuclear invasion indicates the activity of an inflammatory process [21] and is supposedly symptomatic. It is suspected that patients with chronic duodenitis are the early cases of duodenal ulcer characterized by dyspeptic symptoms, only with normal macroscopic appearance [7]. However, studies do not show significant association between chronic duodenitis and dyspepsia in children [16, 26], which is similar to what we see in our study.

Almost all children had normal endoscopic findings. EED is a sub-acute inflammatory condition of the small intestinal mucosa [18], which is expected to show microscopic alterations only visible by histopathology. Any change in the macroscopic appearance of the gut which is expected to be visible in naked eye by endoscopy would be highly unlikely.

Recent studies suggest that SIBO is common among children of the developing world and 16.7% of children less than 2 years in Bangladesh are SIBO positive. This figure conforms well to our findings. The frequency of SIBO is found to be low among patients with dyspepsia [27]. This also correlates with our findings. Intestinal parasitic infection is also considered to be a cause of dyspepsia in children and the prevalence of intestinal parasitic infections in Bangladesh is also high. However, the children studied were also found negative for parasitic infection. It might be due to the fact that anti-helminthic therapy was provided to all the children as per national guidelines. The percentage of children positive for HpSA was low (14.8%). A study conducted in 1997 on children residing in a poor peri-urban community of Bangladesh showed that the prevalence of *H. pylori* infection at age10–15 months was 33% while at 5–8 years was 84% [28]. The overall prevalence of *H. pylori* infection is strongly correlated with socioeconomic conditions [29]. With the improvement of these conditions during these years might have resulted in a decrease in the rates. In addition, children of the current study belong from an urban slum compared with those residing in a poor peri-urban community setting in the previous study, which might also have played a role for the reduced rates. A recent study showed that, the organisms are not usually detected in the first years of life, followed by very high seroconversion rates [30], which supports the findings of both the previous study and the current study, as the mean age of the children of our current study was 18 ± 2 months.

Children positive for *H. pylori* infection had significant association with dyspepsia. Results of a meta-analysis showed that the prevalence of *H. pylori* infection was greater in dyspeptic adults with an odds ratio of 2.3 (95% CI 1.9–2.7) [13]. A study in Bangladesh showed that more than half of the dyspeptic patients are having *H. pylori* infection [31]. However, there is paucity of data regarding the association between dyspepsia and *H. pylori* infection in young children from this region. Studies done on children in Middle-Eastern countries found that the prevalence of *H. pylori* infection was high among children having dyspeptic symptoms [32, 33].

Keeping trend with the previous studies [17, 34], a poor correlation between endoscopic and histological diagnosis of duodenitis in children was observed in this study as well. The negative predictive value of the normal duodenal mucosa is 81.5%, meaning that an apparently normal-looking mucosa might have some mild form of pathological changes, resulting in unsuspected pathology mostly in asymptomatic patients [25]. Long *et al.* [34] reported 54% sensitivity in estimating histological duodenitis with endoscopic findings in children, whereas Alper *et al*. [17], showed a lower sensitivity of 37%. Our data show an even lower sensitivity of 19%, which might have been linked with EED in these children, highlighting the importance for duodenal biopsies even in the event of normal appearing duodenal mucosa during upper GI endoscopy. This might help to minimize overlooking certain GI disorders, as well as CD or EED, and benefit thereof by appropriate treatment or intervention.

### Limitations

This study was conducted as a part of the BEED study and the target population of the present study were stunted (LAZ < −2) and at risk of stunting (LAZ < −1 to −2) children aged between 12 and 24 months. As collection of small intestinal biopsy samples from absolutely healthy, well-nourished children without an evident clinical condition is ethically infeasible, the findings could not be compared with an absolutely healthy control group. Association of EED markers with gut histology was also not explored. This remained a limitation of the study.

## CONCLUSION

The number of children with chronic duodenitis was found to be significantly high, majority of them being asymptomatic. Children positive for HpSA had significant association with dyspeptic symptoms. A poor correlation was however, observed between endoscopic and histological diagnosis of duodenitis. Further studies are warranted to monitor the development of pathology or symptoms in these young children.
